# Supplementary material for: Resolving the full spectrum of human genome variation using Linked-Reads
Source: Genome Res. 2019 Apr;29(4):635–45. doi: 10.1101/gr.234443.118 (PMC6442396; doi:10.1101/gr.234443.118)
Supplement: Supplemental Material [file supp_gr.234443.118_Supplemental_longranger-2.2.zip › longranger-2.2.tar.bz2!/lr2.2-public/lib/stan/cmdstan/stan/doc/api/html/ps__point_8hpp_source.html]

Stan: src/stan/mcmc/hmc/hamiltonians/ps\_point.hpp Source File


|  |  |
| --- | --- |
|  | Stan  2.5.0  probability, sampling & optimization |


- Main Page
- Related Pages
- Namespaces
- Classes
- Files

- File List
- File Members

All Classes Namespaces Files Functions Variables Typedefs Enumerations Enumerator Friends Macros Pages

- src
- stan
- mcmc
- hmc
- hamiltonians

ps\_point.hpp

Go to the documentation of this file.

1 #ifndef STAN\_\_MCMC\_\_PS\_POINT\_\_BETA

2 #define STAN\_\_MCMC\_\_PS\_POINT\_\_BETA

3

4 #include <fstream>

5 #include <string>

6 #include <boost/lexical\_cast.hpp>

7

8 #include <vector>

9 #include <stan/math/matrix/Eigen.hpp>

10

11 namespace stan {

12

13  namespace mcmc {

14

15  // Point in a generic phase space

16  class ps\_point {

17  friend class ps\_point\_test;

18

19  public:

20

21  ps\_point(int n): q(n), p(n), V(0), g(n) {};

22

23  ps\_point(const ps\_point& z): q(z.q.size()), p(z.p.size()), V(z.V), g(z.g.size())

24  {

25  fast\_vector\_copy\_<double>(q, z.q);

26  fast\_vector\_copy\_<double>(p, z.p);

27  fast\_vector\_copy\_<double>(g, z.g);

28  }

29

30

31  ps\_point& operator= (const ps\_point& z)

32  {

33

34  if(this == &z) return \*this;

35

36  fast\_vector\_copy\_<double>(q, z.q);

37

38  V = z.V;

39

40  fast\_vector\_copy\_<double>(p, z.p);

41  fast\_vector\_copy\_<double>(g, z.g);

42

43  return \*this;

44

45  }

46

47  Eigen::VectorXd q;

48  Eigen::VectorXd p;

49

50  double V;

51  Eigen::VectorXd g;

52

53  virtual void get\_param\_names(std::vector<std::string>& model\_names,

54  std::vector<std::string>& names) {

55  for(int i = 0; i < q.size(); ++i)

56  names.push\_back(model\_names.at(i));

57  for(int i = 0; i < q.size(); ++i)

58  names.push\_back(std::string("p\_") + model\_names.at(i));

59  for(int i = 0; i < q.size(); ++i)

60  names.push\_back(std::string("g\_") + model\_names.at(i));

61  }

62

63  virtual void get\_params(std::vector<double>& values) {

64  for(int i = 0; i < q.size(); ++i)

65  values.push\_back(q(i));

66  for(int i = 0; i < q.size(); ++i)

67  values.push\_back(p(i));

68  for(int i = 0; i < q.size(); ++i)

69  values.push\_back(g(i));

70  }

71

72  virtual void write\_metric(std::ostream\* o) {

73  if(!o) return;

74  \*o << "# No free parameters for unit metric" << std::endl;

75  }

76

77  protected:

78

79  template <typename T>

80  static inline void fast\_vector\_copy\_(Eigen::Matrix<T, Eigen::Dynamic, 1>& v\_to,

81  const Eigen::Matrix<T, Eigen::Dynamic, 1>& v\_from) {

82  int sz = v\_from.size();

83  v\_to.resize(sz);

84  if (sz > 0) std::memcpy(&v\_to(0), &v\_from(0), v\_from.size() \* sizeof(double));

85  }

86

87  template <typename T>

88  static inline void fast\_matrix\_copy\_(Eigen::Matrix<T, Eigen::Dynamic, Eigen::Dynamic>& v\_to,

89  const Eigen::Matrix<T, Eigen::Dynamic, Eigen::Dynamic>& v\_from) {

90  int nr = v\_from.rows();

91  int nc = v\_from.cols();

92  v\_to.resize(nr, nc);

93  if (nr > 0 && nc > 0) std::memcpy(&v\_to(0), &v\_from(0), v\_from.size() \* sizeof(double));

94  }

95

96  };

97

98  } // mcmc

99

100 } // stan

101

102

103 #endif

stan::mcmc::ps\_point::get\_params

virtual void get\_params(std::vector< double > &values)

**Definition:** ps\_point.hpp:63

stan::mcmc::ps\_point::operator=

ps\_point & operator=(const ps\_point &z)

**Definition:** ps\_point.hpp:31

stan::mcmc::ps\_point::fast\_vector\_copy\_

static void fast\_vector\_copy\_(Eigen::Matrix< T, Eigen::Dynamic, 1 > &v\_to, const Eigen::Matrix< T, Eigen::Dynamic, 1 > &v\_from)

**Definition:** ps\_point.hpp:80

stan::mcmc::ps\_point::ps\_point

ps\_point(int n)

**Definition:** ps\_point.hpp:21

stan::mcmc::ps\_point::write\_metric

virtual void write\_metric(std::ostream \*o)

**Definition:** ps\_point.hpp:72

stan::mcmc::ps\_point

**Definition:** ps\_point.hpp:16

stan::mcmc::ps\_point::get\_param\_names

virtual void get\_param\_names(std::vector< std::string > &model\_names, std::vector< std::string > &names)

**Definition:** ps\_point.hpp:53

stan::mcmc::ps\_point::V

double V

**Definition:** ps\_point.hpp:50

stan::mcmc::ps\_point::q

Eigen::VectorXd q

**Definition:** ps\_point.hpp:47

stan::mcmc::ps\_point::p

Eigen::VectorXd p

**Definition:** ps\_point.hpp:48

stan::mcmc::ps\_point::fast\_matrix\_copy\_

static void fast\_matrix\_copy\_(Eigen::Matrix< T, Eigen::Dynamic, Eigen::Dynamic > &v\_to, const Eigen::Matrix< T, Eigen::Dynamic, Eigen::Dynamic > &v\_from)

**Definition:** ps\_point.hpp:88

stan::mcmc::ps\_point::g

Eigen::VectorXd g

**Definition:** ps\_point.hpp:51

Eigen.hpp

stan::math::size

int size(const std::vector< T > &x)

**Definition:** size.hpp:11

stan::mcmc::ps\_point::ps\_point

ps\_point(const ps\_point &z)

**Definition:** ps\_point.hpp:23

stan::mcmc::ps\_point::ps\_point\_test

friend class ps\_point\_test

**Definition:** ps\_point.hpp:17


---

[ Stan Home Page ]

*© 2011–2014,
Stan Development Team.*
